# Extracting Abscessed Teeth

**Published:** 1907-08-15

**Authors:** G. B. Mitchell


### EXTRACTING ABSCESSED TEETH.

BY G. B. MITCHELL,

Abstract of paper published in July Cosmos.

It has been the experience of the writer, as well as that of others, to hear some one remark that he would not extract a tooth during an attack of incipient pericementitis—while the battle was being fought between the leucocytes and the invading bacteria, and before infiltration of the soft tissues had begun. One wonders at the reason for not extracting. Our worthy *confrere*, upon being asked, answers, "Dangerous." "But why dangerous?" you ask, and the reply will be either an indefinite one or a French hunch.

Others will tell you or the young practitioner that they never hesitate to extract a tooth at any stage of alveolar infection or inflammation. In the face of so much uncertainty, what should one do and believe? The author knows a score of cases where the attending dentist, and even the physician, refused to extract, and yet when asked for reasons could offer no logical ones.

Such diversity of opinion on this matter and such indicision as to procedure was precipitated upon a meeting of a meeting of the Buffalo City Dental Association last winter, and the writer then and there determined to find out if at all possible, what, if any, danger (and why) could accompany the extraction of teeth the seat of abscesses. The opinions expressed by various authors and journals, and the replies to letters written to certain members of high standing in the dental profession constitute, in a general sense, the present paper.

Attention was called to the former stand of non-extraction assumed by Dr. Robt. T. Morris, professor of surgery in the New York Post-Graduate Medical School, who in a recent article entitled "Infection of the Lymph-glands of the Mouth and Throat" (*Cosmos*, June 1904), says:

One class of infections, very dangerous ones, have been frequently overlooked by dentists. . . . . These are infections following the removal of abscessed teeth. Patients die and the cases are not reported; they come to the hospital to be treated for pneumonia. There are patients dying this minute in this city from the result of having abscessed teeth extracted while in the course of acute infection; there are cases dying continually . . . . not recorded and not discussed, for the reason that they are entered at our hospitals as cases of pneumonia; but they are cases of septic pneumonia, embolic pneumonia, resulting from infection from abscessed teeth. Very often the dentist knows nothing about it. He removes a tooth; he hears, four or five days later, that the patient has developed pneumonia—believes it to have been a coincidence, thinks his part in the case is not one of consequence, and the patient dies. The case is recorded as a death from pneumonia—not as septic pneumonia from an abscessed tooth.

The writer finds that dental literature is sadly lacking in reports on cases of infection and death due to extraction, but this deficiency may in no way indicate that such conditions have not occured, or do not occur at the present time. More nearly is this due to our short-sightedness in matters pertaining to pathogenesis.

According to Vignal and Suckdorf, an adult man passes from thirty to fifty billion of bacteria daily in the feces. Many are harmless in the healthy individual, but, as will later be shown, their virulence is felt after accidents and injuries, such as gunshot wounds, injuries in extraction, shock, catarrhal conditions of the mucous membrane, etc. One is in constant danger of being infected. It has been scientifically demonstrated that every pathological lesion in the the oral cavities is due to the attacks of micro-organisms. Our resistance to these micro-organisms depends on the resistance of the tissues to pathological influences, and varies in direct ratio to the degree of health of the individual.

Many undoubtedly remember Dr. Millers' surprising statement of a year or so ago, when he said that the bite of a girl was as deadly as the sting of a venomous serpent.

Kirk (*Dental Cosmos*, November 1900) shows a culture of the diplococcus of pneumonia from Miller's specimens, which was found in the blood of a mouse which had been inoculated with a culture from the saliva, thus showing that the blood-stream is a carrying agent for pathogenic organisms. They are undoubtedly harmless until they reach some point of diminished resistance, and there set up inflamation. This point of diminished resistance in our case is the diseased tooth, and the shock or injury of extraction throws the diplococcus into a state of activity.

In four cases where he secured specimens of the pus from pericemental abscess, the bacteriological investigation showed in each the presence of the diplococcus pneumoniae. A rabbit inoculated with the pure culture of this diplococcus died in ten days, with all the symptoms of the peculiar form of toxemia which is produced by inoculation with the diplococcus pneumoniae.

J. Leon Williams (*Dental Cosmos*, April 1899) reports having found organisms of a diphtheroid character present in a large majority of the mouths he examined, and often where there was no history of diphtheria. It is not the so-called "catching" of a disease, as we formerly thought, but the continued presence of these virulent pathogenic organisms in the oral cavity, which gaining control over us when physically below par—as in accidents, injuries, etc.—induce pathological disturbances.

Robert T. Morris says: "We see cases in which teeth have been removed while there is an abscess of the tooth in an acute, progressive stage, when the abscess is forming. The bacteria are in a state of active proliferation—very active development. If a very small fragment of the bone be broken, the veins of the cancellous structure of the bones fill with thrombi, which, becoming infected, become emboli, and embolic infections follow three or four days after the accident."

D. J .Brown says that healthy oral cavities and their adnexa are especially exempt from infectious processes following injuries, while an oral cavity which is septic from an abscessed tooth-traumatism without preliminary and after-treatment is very dangerous, on account of the possibility of extension of the suppuration to some distant part. If a number of micrococci gain entrance into the bottom of the wound, they at once multiply, using the blood-clot and its extensions into the bloodvessels, together with the adjacent dead tissues, as a welcome soil for their development.

He cites the following case: A young lady of twenty-three. History, abscessed tooth of six months' standing, accompanied by swelling, chills, and fever. Tooth extracted without preliminary treatment. Infection manifested itself in two days throughout the neck, chest, abdomen, and anterior spine of the ilium. Diagnosis, abscess in several places in the body, necessitating several operations, patient finally dying of general toxemia. Dr. Brown says: "Here is a case of secondary infection through the lymphatics from the oral cavity, in which a young and vigorous woman lost her life from lack of preliminary and post-operative treatment for the removal of an abscessed tooth." His other cases show how readily the lymphatics carry bacteria and toxins.

Dr. John Jesensky, of Prague, reports, in the *Dental Cosmos* for July, 1901, a case of suppurative inflammation of the upper jaw. Alveolar periostitis was present, and a painful and forceful extraction resulted in an infectious periostitis and osteomyelitis, which spread rapidly through the upper alveolus.

Thus we see that the danger of septicemia is ever present in extraction, as it is very difficult to keep aseptic a mucous surface, especially a blood-clot, that is constantly bathed in septic matter and pathogenic micro-organisms. Septic blood conditions have been observed in pyorrhea of the nat-ture of those occuring in carcinoma.

D. C. Sabatier in *The Cosmos*, June 1904, says: "It has been observed that serious complications follow occasionally lesions of the teeth and suppuration of the gingivo-dental region and stomatitis, owing to a systemic intoxication by varieties of microbes or their toxins. . . . These intoxications, which belong to the type of septicemiae, may be of the following varieties: (a) Chronic septicemiae, almost always consecutive upon prolonged suppurations of the gingivo-dental region. (b) Acute septicemiae which are stages in the course of chronic septicemiae or else true acute attacks following surgical interventions or other causes render the organism more vulnerable. . . . In the majority of cases, the progress of acute septicemiae is fatal. . . . The possibility of severe intoxications following suppuration of the gingivo-dental region explains the necessity of combating by all the means at our disposal any suppuration, whatever be its degree of intensity."

Morris says that as a general principle, the greatest danger of surgical intervention is at a time when bacteria are rapidly proliferating. He has seen two or three cases of infections in the course of a week, resulting from the removal of teeth the seat of acute alveolar abscess. And he is quoted as saying that there is no operation in minor surgery that is so fatal as the extraction of lower molars, especially the third molars the seat of acute alveolar abscess.

C. A. Harmann, M.D., reports three cases of suppurating sockets of teeth, resulting in submaxillary and parotid infection, followed by septic thrombosis of the cavernous sinuses and death. His explanation of the path the infectious process follows in these cases of extension from the teeth to the cavernous sinuses is interesting.

He also cites a case of fatal sepsis following the extraction of a molar upon the pericementum of which an abscess had developed. Death was due to thrombosis in the cavernous sinus.

Inflammatory phenomena resulting from infection of the alveolus subsequent to the extraction or by the retention of the abscess sac are usually accompanied by pain. Infection in such case may follow from three to four days after the extraction.

In the *Dental Cosmos* for October 1906 is an abstract from *Semaine medicale,* giving a case of death following the extraction of a tooth under cocain and adrenalin anesthesia, the case having been reported by Dr. Maragliano, of the Faculty of Medicine, Genoa, Italy. The tooth was extracted owing to a severe attack of alveolo-dental periostitis. On the following day there was intense fever, and in addition the gingival tissue presented the appearance of having become the seat of an active necrotic process. Symptoms of intense purulent infection soon appeared, and culminated in the death of the patient, which occured eight days after the extraction. The reviewer (Cosmos, page 1061) says: "Although the extraction of a tooth during or shortly after an acute attack of pericemental infection is *per se* ample cause to account for a fatal termination, Dr. Maragliano opines that the accident might have been averted had the extraction been performed without the therapeutic assistance of the cocain-adrenalin solution. To the reviewer such a view of the case seems hardly plausible, The nucleus of infection was present before the injection was performed, which at the utmost could only have intensified the inflammatory process. . . . Fatal cases traceable to pericemental infections are not so rare as the author seems to imply, and many just such cases as the one here reported can be found recorded in medical and dental literature. The severity of the symptoms and the fact that death did not occur until a week after the extraction point strongly to septicemia of dental origin as the paramount cause of the fatal termination."

Dr. Low, in discussing Dr. Morris' paper, before quoted thought that the difference of opinion as to the immediate extraction of the abscessed teeth might possibly come from the fact that in private practice we see a different class of patients from that of a hospital clinic, the latter being, as a rule, physically below par. But even if grave danger be coimminent with extractions only in public clinics, we should not dismiss the subject lightly. Anything less than our best efforts may result in severe injury. We all have the physically deficient in our practices, rich and poor alike.

The following questions were submitted to a number of dentists and their replies are given below.

Do you (or did you) ever hesitate to extract in cases of pericementitis and alveolar abscess, or any acute alveolar infection?

If so, in what instances and for what reasons?

Do you differentiate in extraction between pericemental or alveolar infections caused by colds and those due to other causes?

In your experience, do you think there is any danger (as per extract) attending extraction at any stage of alveolar infection?

If so, what dangers, and for what reasons?

---

I suppose this is a continuation of the discussion that has been going on for years, certain writers and clinicians opposing the extraction of teeth during the acute periods of alveolar abscess. My experience and observation seem to warrant me in saying that whenever dangerous symptoms come up in the course of alveolar abscess, the most certain way of abating them is to at once extract the teeth concerned. In saying this, I do not wish to infer that this will in every case relieve the symptoms or bring about a radical cure of the case, but the rule is, that it is the safest procedure. It is usually the continuation of severe inflammatory and suppurative processes that causes wide necrosis of bone and septicemia, particularly, is the most dangerous feature of alveolar abscess.

As to pneumonia occuring from these infections, I have no knowledge, having never seen a case, and should not say that such a thing is impossible; yet taking all the circumstances into consideration, it does seem rather improbable. I have had pesronal knowledge of three cases of abscess of the lungs resulting from the injection of carbolic acid for the radical cure of piles. In these cases the carbolic acid was injected into a mass of loose and very vascular tissue; probably coagula at once entered the veins and were conveyed to the heart, and from the heart to the lungs, where they lodged. The chances for such an occurrence from alveolar abscess are not nearly so good as they would be from such tissue as that in which the carbolic acid was injected. In these cases of alveolar abscess the tissues are filled with the inflammatory products, and the vessels for the most part are closed, giving conditions which are exactly the opposite of the cases I have stated, in which the floating away of coagula in the blood-streams would be least probable, and certainly would occur least often. While this seems to me the case, it may be possible that I have overlooked cases of pneumonia—or so-called pneumonia—resulting from the transplantation of micro-organisms to the lungs, but it is difficult to think that this should occur in any considerable number of cases without there having been more explicit observations of them.

In your fifth question you ask, "Do you think there is any danger attending extraction, at any stage of alveolar infection?" I will say in answer to this that there is often danger of septicemia resulting from alveolar infection. I think that the extraction of the teeth in these conditions is the best way to avert the danger, though it may not always be successful. As to the general subject, I will say that there are quite a number of deaths occuring from infections in connection with alveolar abscess that are not being reported as such. So far as I am able to judge from my own observations, they are all deaths from septicemeia. Several have occured in Chicago, and I have reports of a number of others; in my practice death has been very narrowly averted in a number of cases. All of these have taken on the phase of septicemia, and in none of them, that I know of, has lung trouble of any kind formed a principal item in the conditions found. The *danger to life* from alveolar abscess is not at all sufficiently recognized, and in my opinion deaths occuring from this cause should be carefully reported with all the attendant symptoms, until such time as the dental profession, and the medical as well, recognize these dangers and the particular directions that the malady induced takes.—*G. V. Black.*

It has been pretty clearly made out through bacteriological researches, first, that the pneumococcus is a nearly constant inhabitant of the human mouth; some authorities assert that in as high as eighty per cent. of human mouths the pneumococcus is to be found; and it is still pretty clearly made out by laboratory research that the pneumococcus is one of the principal exciters of inflammatory conditions in the pericemental membrane, and that the infection of the pericemental membrane may, and usually does, take place via the pulp-canal, when that canal is exposed; and that it may take place through the blood-current, or possibly through the gingival margin, in cases where the pulp-canal is not open, but where we have that type of abscess which has been called pericemental abscess—that is to say, abscess upon teeth with living pulps. The pneumococcus is apparently the exciter of the initial pericemental inflammation, and when the inflammation is once lighted up, the bacteria of pus-formation invade the tissue, forming a true abscess. Under these conditions, as you will see, an inoculation of the tissues by the pneumococcus has occured even before the tooth is extracted, and it is questionable to my mind that the extraction of a tooth under conditions of active inflammation from such an infection adds one iota of danger to the subsequent infection of the lung. On general principles I am of the opinion that it is good surgery to remove such abscessed tooth, because its infected pericemental membrane is the focus of inflammation, and because in the majority of cases the removal of a tooth which is the focus of infection tends to relieve the inflammation; or, otherwise stated, on general principles I am of the opinion that in cases where a secondary pulmonary infection occurs, it would happen equally whether the tooth were extracted or not. It is good surgery and rational therapeutics to remove the source of infection in all cases, and I cannot see that the retention of the tooth would reduce the chances of the pulmonary infection in any case where it was likely to occur from a dental origin.

I never have known of a case where I could reasonably trace out a connection between a dento-alveolar abscess and subsequent pneumonia. I have, however, seen cases of extensive necrosis of the lower jaw, with exfoliation of a considerable portion of the alveolar border and loss of a number of sound teeth, due to infection from the pneumococcus, an where that organism was definitely determined by well-known research methods to have been the exciter of the necrotic inflammation, and where the original point of entry was through the open canal of a carious tooth.

You will see from the foregoing that I cannot take the ground that there is danger in the extraction *per se*. I should no more hesitate to extract an infected tooth than I would to remove a splinter which was the carrier of infection and cause of suppuration somewhere in the tissues of the body. There is a very palpable danger in cases of extraction during abscess of a tooth that the extractor may be charged with causing the infection, because the removal of a tooth does not, in all cases, cure the abscess.—*E. C. Kirk.*

I am glad to see attention called to the more serious aspect of diseases of the teeth and alveolar structures in relation to fatal results. My work being almost wholly confined to hospital cases, such conditions naturally come under my observation more than ordinarily would be the case in office practice; and I have constantly endeavored, during the past few years, to bring forward the idea of the fatal possibilities that lie directly in line with many pathological affections that dentists commonly undertake to treat. As a matter of fact, the pneumococcus and its direct results are only a part of the pathogenic enemies to be feared. How many lives dentists could have saved with a better knowledge of pathology will never be known.

One must differentiate between acute and chronic conditions; between those in which there is general manifestation of toxic influences; between cases in which large gatherings of pus can readily be evacuated without exposing freshly open vessels to infection, as might be the case under immediate extraction, and between those patients in whom blood examinations hows marked deficiency of those elements immediate extraction, and between those patients in whom blood examination shows marked deficiency of those elements upon which dependence must be placed in battling against septic dangers. Ofted the question is very difficult one to decide, *i. e.*. whether the danger is greater in allowing the direct cause of irritation and infection to remain while other measures are employed to give relief, instead of resorting to immediate extraction, or whether the complete removal of the exciting cause would give more prompt relief, and in the end be safer.

A man came to me for operation at my clinic at the University of Iowa. I refused to operate unless he would delay it long enough to enable blood, urine and other examinations to be made. He went to a general surgical clinic in order to have immediate operation for the cure of what appeared to be a disturbance of the lower incisors, with necrosis of the surrounding alveolar structures. A general surgeon undertook the case and performed the operation at once, with the result that the patient died upon the operating table.

Undoubtedly, in vast numbers of cases with conditions of otherwise reasonably good health upon the part of infected individuals, the most prompt and efficient method of giving relief is by removal of the cause of infection, whether it be tooth or alveolar structures; but when the blood examination shows that the hemoglobin is lower than normal, and the blood corpuscles are disarranged in their numerical proportions to a degree indicative of disease; or when temperature and pulse or urine or bacterial conditions present indications of serious pathologic disturbance, the question of these or other physical signs of diminished bodily resistance must be weighed carefully against the local demands for immediate intervention. It is my invariable practice wherever possible, to have such examinations made in cases where the symptoms indicate a rather serious condition. This has been a great safeguard to me, and I know of no other means by which one may safely arrive at a true understanding of individual conditions upon which a decision as to the method of procedure must be based.—

*G. V. I. Brown.*

In reply to your inquiries regarding extraction of teeth in the presence of pericementitis and alveolar abscess, in my opinion the tooth should be removed, and I should not hesitate to do so. One trouble of the present status is that a number of men send extracting cases to a specialist. The dentist seems to think that his care of the case ceases with the recommendation. On the other hand, the specialist too often dismisses the patient after extracting the tooth and receiving his fee. The result is that the wound receives no after care. In my practice, when I send a patient to a specialist for the removal of a tooth, I either have it definitely understood that he is to continue the treatment of the case until the wound is healed, or I take charge of that portion of the work myself. In other words, the removal of a tooth, especially in the presence of infection, demands the same surgical after-care as would be accorded to similar conditions elsewhere in the body.—*R. Ottelengui.*

(1) That there is infinitely more danger in leaving an abscessed tooth in its socket too long than there can be in its removal.

(2) I should never hesitate to extract in cases of pericemental or alveolar abscess, providing I could not by other means restore the surrounding tissues to a normal condition.

(3) Causes of all pus-formation should be well and carefully differentiated. If due to a local affection, cure can certainly be much easier obtained than if it were due to a systemic diathesis.

(4) I can see no danger in extraction; it means in most cases simply the removal of the cause— the rational treatment of all diseases.

Septic pneumonia, embolic pneumonia, if at all due to abscessed teeth, are not due to the extraction of the diseased teeth *per se*. They are due to the fact that the teeth were not extracted in time, thus allowing the pus to be absorbed, and to produce a remote septic condition.—*R. H. Hofheinz.*

I have seen more more harm result from the advocacy the retention of abscessed teeth than from their prompt removal. Death in such cases occurs in spite of the removal of the cause of infection, rather than because of it. Teeth are retained too long a period in an attempt to await the passing of the acute stage, during which time the suppuration makes more progress, and the system of the patient becomes undermined. Septicemia and pyemia are the natural results of prolonged retention of pus. It is an interesting observation that Dr. Morris has made, that such cases exhibit evidences of embolic pneumonia.

I never hesitate to extract a tooth because of an acute infection, if I am reasonably sure that the tooth should be sacrificed. If an acute abcsess can be induced to subside, and the tooth conserved by an incision of the gum tissue or a puncture of the process, I believe this to be the best means of dealing with abscesses during the acute stage; not because I fear the consequences of taking out the tooth, but rather to save what may be a valuable masticating organ. To say that "the extraction of a tooth during the active process of inflammation or suppuration is without danger" would be a mis-statement, unless it were added, "providing the case was handled as the careful surgeon would treat any other inflamed or infected wound." Complications will arise in the extraction of teeth so long as operators fail to realize the importance of dealing with this operation as similar conditions are handled in other parts of the body.

*M. J. Schamberg.*

There has been, and there is today, a general lack of sterilization of the oral cavity before and after extraction. To the knowledge of the writer, no anti- or post-extraction sterilization of the mouths of the patients is insisted upon in our colleges. In the eight years of his dental experience he has yet to see the practitioner who sterilizes instruments and field of operation before and after extraction. To neglect this important operation is criminal malpractice. One must instruct the patient as to the proper care after extraction. The extraction of a diseased tooth surrounded by diseased tissues is a source of great danger to the patient. If you send the latter to a specialist, see to it that either the specialist or yourself treats the wound as a surgeon would. Patients are more lax in returning after extraction than after any other operation.

The idea that the saliva is in any way germicidal is an erroneous view, as has been brought out by Miller's experiments. They have established the fact that neither the oral fluids nor their separate constituents have the power to arrest or even perceptibly retard the growth of bacteria.

Aseptic surgery is the only surgery known today by men of established reputation. It is just as true of the dental as of medical work. Those who adopt it least will always be the last.

We must see that it is often a very serious affair to extract teeth the seat of pericemental infection; that there is danger of systemic infections and death, and that the determination of the cause of the infection is a matter of careful diagnosing. When an infection is developing in the alveolus or contiguous membranes, the micro-organisms proliferate rapidly, and we should be careful not to injure or shock the focus of infection before the period of active phagocytosis. The proper time to extract, if extraction be found advisable, is when either side, bacteria or leucocytes, is shown to have been the victim, as is evidenced either by tissue infiltration on one side (bacteria winning) or diminished periostitis on the other (leucocytes winning).

Let us do away with any possibility of infections by cautious diagnosis and aseptic surgery.

